# Differences in clinical characteristics and outcomes between patients with grade 3a and grades 1–2 follicular lymphoma: a real-world multicenter study

**DOI:** 10.1186/s40364-023-00462-z

**Published:** 2023-02-06

**Authors:** Jie Zha, Qinwei Chen, Jingjing Ye, Haifeng Yu, Shuhua Yi, Zhong Zheng, Wei Xu, Zhifeng Li, Lingyan Ping, Xiaohua He, Liling Zhang, Caixia Li, Ying Xie, Feili Chen, Xiuhua Sun, Liping Su, Huilai Zhang, Liyuan Fan, Zhijuan Lin, Haiyan Yang, Weili Zhao, Lugui Qiu, Zhiming Li, Yuqin Song, Bing Xu

**Affiliations:** 1 Department of Hematology, The First Affiliated Hospital of Xiamen University and Institute of Hematology, School of Medicine, Xiamen University, Xiamen, 361003 P.R China; 2 Key Laboratory of Xiamen for Diagnosis and Treatment of Hematological Malignancy, Xiamen, China; 3 Department of Hematology, Qilu Hospital, Cheeloo College of Medicine, Shandong University, Jinan, China; 4 Department of Lymphoma, Cancer Hospital of the University of Chinese Academy of Sciences (Zhejiang Cancer Hospital), Hangzhou, China; 5 Department of Lymphoma, Institute of Cancer and Basic Medicine (IBMC), Chinese Academy of Sciences, Hangzhou, China; 6 State Key Laboratory of Experimental Hematology, National Clinical Research Center for Blood Diseases, Blood Diseases Hospital & Institute of Hematology, Chinese Academy of Medical Sciences & Peking Union Medical College, Tianjin, China; 7 Shanghai Institute of Hematology, State Key Laboratory of Medical Genomics, National Research Center for Translational Medicine at Shanghai, Ruijin Hospital Affiliated to Shanghai Jiao Tong University School of Medicine, Shanghai, China; 8 Department of Hematology, The First Affiliated Hospital of Nanjing Medical University, Jiangsu Province Hospital, Nanjing, China; 9 Key Laboratory of Carcinogenesis and Translational Research (Ministry of Education), Peking University Cancer Hospital & Institute, Beijing, China; 10 Department of Medical Oncology, Sun Yat-Sen University Cancer Center, Guangzhou, China; 11 State Key Laboratory of Oncology in South China, Guangzhou, China; 12 Collaborative Innovation Center for Cancer Medicine, Guangzhou, China; 13 Cancer Center, Union Hospital, Tongji Medical College, Huazhong University of Science and Technology, Wuhan, China; 14 National Clinical Research Center for Hematologic Diseases, Jiangsu Institute of Hematology, The First Affiliated Hospital of Soochow University, Suzhou, China; 15 15Shengli Clinical Medical College of Fujian Medical University, Department of Hematology, Fujian Provincial Hospital, Fujian Medical University, Fuzhou, China; 16 Lymphoma Division, Guangdong Provincial People’s Hospital, Guangdong Academy of Medical Sciences, Guangzhou, China; 17 Second Hospital of Dalian Medical University, Dalian, China; 18 Shanxi Province Cancer Hospital/ Shanxi Hospital Affiliated to Cancer Hospital, Chinese Academy of Medical Sciences/Cancer Hospital Affiliated to Shanxi Medical University, Taiyuan, China; 19 Department of Lymphoma, Tianjin Medical University Cancer Hospital, Tianjin, China

**Keywords:** Follicular lymphoma, Histological grading, Clinical feature, survival, Histological transformation

## Abstract

**Background:**

The difference between clinical characteristics and outcomes between follicular lymphoma grade 1–2 (FL1-2) and FL3a defined pathologically remains unclear, resulting in uncertainty how to treat FL3a. However, it may be crucial for clinicians to discriminate grade 3a and grade 1–2 for predicting prognosis and thus making treatment decisions.

**Methods:**

We compared 1403 patients with FL1-2 and 765 patients with FL3a diagnosed between January 2000 and December 2020 from fifteen centers nationwide in China to describe differences in clinical characteristics and outcomes.

**Results:**

Compared with FL1-2 patients, FL3a subgroup had a higher percentage of elderly patients (*P* = 0.003), and relatively more FL3a patients presented with increased levels of LDH (*P* < 0.0001) and higher Ki-67 indexs greater than 30% (*P* < 0.001). More FL3a patients were treated with CHOP ± R (*P* < 0.0001), and fewer were treated with the watchful-waiting approach (*P* < 0.0001). The results showed a higher incidence of relapse among FL3a patients, in which more patients underwent histological transformation (HT) when compared to FL1-2 (*P* = 0.003). 1470 (76.2%) patients of the entire cohort received R-CHOP therapy; survival analysis revealed that FL3a patients had a worse progression-free survival (PFS) rate than FL1-2 patients. Survival of FL3a patients with respect to FLIPI showed an inferior PFS in the intermediate and high-risk groups than FL1-2 patients. FL3a patients had a much worse prognosis than FL1-2 with or without progression of disease within 24 months (POD24). FL3a patients had higher likelihood of lymphoma-related death (LRD, *P* < 0.05), whereas the rates for non-LRD were comparable.

**Conclusion:**

In conclusion, this study demonstrates a marked difference in clinical features and outcomes in FL3a patients compared with FL1-2 patients. The results highlight the need for applying therapeutic approaches distinct from FL1-2 when treating FL3a patients.

**Supplementary Information:**

The online version contains supplementary material available at 10.1186/s40364-023-00462-z.

## Background

Follicular lymphoma (FL) is the most common indolent lymphoma and accounts for approximately 30% of all newly diagnosed non-Hodgkin lymphomas in Western countries [1]. FL is classified into three distinct pathologic grades (i.e., FL1-3) according to the number of centroblasts per high-power field [2]. FL3 can further be subdivided into FL3a and FL3b in the current WHO classification. In the 5^th^ Edition WHO classification, follicular large B-cell lymphoma (FLBL) mainly refers to FL3b [3], which is considered to be biologically and clinically similar to diffuse large B-cell lymphoma (DLBCL) and managed as such [4]. However, there is less certainty regarding the clinical course and management of patients with FL3a. Some studies suggest that, in patients, FL3a is similar to FL1-2 with an indolent clinical course [5, 6], while others suggest that it has a clinical course similar to FL3b, which is more aggressive [7, 8]. In this context, the NCCN guidelines recommend that some patients be treated for FL and others be treated for diffuse large B-cell lymphoma (DLBCL) [9]. However, which patients with grade 3a should be treated as “low-grade” FL or DLBCL remains poorly defined.

Due to high clinical heterogeneity [10], FL patients are currently managed using individualized strategies [11]. In general, the management of patients with FL1-2 depends on the disease stage and symptoms at presentation. Asymptomatic patients with low tumor burdens often approached with a watchful-waiting strategy [12], whereas symptomatic patients need to be treated, preferably with immunochemotherapy [13, 14]. The regimen combining rituximab and the alkylating agent bendamustine (BR) represents a better therapeutic option for previously untreated patients with low-grade FL (FL1-2) than other regimens combining rituximab with cyclophosphamide, doxorubicin, vincristine, and prednisone (R-CHOP) or with cyclophosphamide, vincristine, and prednisone (R-CVP) [15, 16]. However, clinical studies of FL3a receiving BR or R-CHOP regimens have yielded conflicting results [17, 18].

The immunohistochemical and genotypic characteristics of FL3a have been examined in detail in a previous study, in which patients with FL3a were seen to present with the same immunophenotypical characteristics as patients with FL1-2 [17]. Several studies have found a difference in survival between patients with FL1-2 and FL3a; however, a survival difference has not been consistently observed [6, 8, 19, 20]. Furthermore, many studies have had small sample sizes and lacked control groups. Additionally, little is known about the difference in the risk of disease-specific mortality and relapse following treatment between FL1-2 patients and FL3a patients. In the criteria for FL pathological diagnosis newly-updated by the WHO, both FL1-2 and FL3a have been considered classical FL [3], further blurring the boundary between these two stages. In this context, it is unclear how to treat FL3a patients in the NCCN recommendations [9]. However, due to their potential differences in the outcomes, it may be crucial for clinicians to discriminate grade 3a and grade 1–2 for predicting prognosis and thus making treatment decisions.

To this end, we performed this multicenter study involving a large cohort of 2168 adults newly diagnosed with grade 1-3a FL in the past two decades in China to provide real-world clinical information about demographic and disease characteristics, treatment patterns, therapeutic responses, clinical outcomes, and causes of death (CODs) related to patients with FL1-2 and FL3a. The aim of this study was to exploit this large-scale data set to examine the characteristics, including treatment, of FL1-2 and FL3a patients and to investigate any variations between them in each category.

## Methods

### Patients

We performed a multicenter retrospective study of patients with newly diagnosed FL between 2000 and 2020 at 15 Chinese medical centers. This study was approved by the institutional review board of all collaborative institutions. A total of 2469 FL patients were initially identified in the study, while 301 patients were subsequently excluded due to being < 18 years old, diagnosed with FL3b, lacking adequate clinical information, or lost to follow-up.

Diagnosis was made by the institutional hematopathology expert review board at each site, while central pathologic review was not performed. Transformation from FL to other forms of lymphoma was confirmed by biopsy. According to the institutional standard of care for FL patients, disease staging, treatment selection, and response assessment were carried out at the discretion of treating physicians.

### Variables and endpoints

The demographic and clinical characteristics of FL patients were assessed at initial diagnosis, including age, sex, performance status (PS), disease stage, histological grade, B symptoms, involved lymph nodes (LNs), extranodal disease, BM involvement, and bulky disease; laboratory examination included tests of serum lactate dehydrogenase (LDH), hemoglobin (HGB), and β2-microglobulin (β2-MG). Treatment response was classified as complete remission (CR), partial response (PR), stable disease (SD), or progression of disease (PD) according to the Lugano criteria [6]. OS was defined as the time from diagnosis to death or last follow-up. PFS was defined as the time from diagnosis to progression, relapse, death from any cause, or last follow-up. RFS was defined as the time from diagnosis to relapse of disease. Cause of death was categorized as lymphoma-related causes (e.g., progression, relapse or transformation), treatment-related mortality (TRM), other causes, or unknown. TRM was further classified as infection, cardiac failure, or respiratory failure. If the cause of death was unclear, the case was discussed between the investigators and classified by consensus.

### Statistical analysis

Clinicopathologic characteristics between patients were compared using Wilcoxon rank-sum tests for continuous variables and X2 tests for categorical variables. Median follow-up was determined by reverse Kaplan–Meier analysis. Kaplan–Meier curve was used to estimate PFS, OS and RFS. Proportional hazards regression was used to estimate the effect of risk factors on PFS and OS, for which the results are presented as hazard ratios (HRs) together with 95% confidence intervals (CIs). *P* values for these analyses were calculated by Wald tests. The cumulative incidence of the competing risks of relapses, the competing risks of death and tests of equality for death between groups were analyzed using the cuminc function from the cmprsk package in R.

## Results

### Baseline patient and disease characteristics of FL1-2 and FL3a

A total of 2469 patients newly diagnosed with FL were included in the final analyses. Among them, 301 patients were excluded due to their ineligibility according to the exclusion criteria described in Patients and Methods. As a result, 2168 patients were enrolled in this study, and a comparison of the baseline information about clinical and disease characteristics in FL1-2 and FL3a patients is shown in Table 1.

**Table 1:** Comparison of patients’characteristics in the grade 1 to 2 and 3a follicular lymphoma

Characteristics	Grade1-2	Grade3a	*P* value
No. of patients	1403	765	543
Age			
Median, years (range)	52 (20–95)	54 (18–91)	
> 60 years	365 (26.0)	245 (32.0)	0.003
Male	696 (49.6)	346 (45.2)	0.06
ECOG			
2–4	211 (16.9)	100 (14.5)	0.19
Missing data	156 (11.1)	78 (10.1)	
Rai stage			
III-IV	1103 (83.2)	519 (70.7)	< 0.001
Missing data	78 (5.5)	31 (4.0)	
B symptoms			
Yes	176 (19.6)	106 (21.7)	0.37
Missing data	504 (35.9)	278 (36.3)	
Lymph nodes > 4			
Yes	838 (65.0)	399 (59.5)	< 0.001
Missing data	114 (8.1)	65 (8.5)	
Extranodal disease			
Yes	615 (55.1)	292 (50.4)	0.07
Missing data	287 (20.4)	186 (24.3)	
Disease bulk > 6 cm			
Yes	275 (23.2)	146 (22.5)	0.77
Missing data	219 (15.6)	117 (15.2)	
Marrow involved			
Yes	457 (34.1)	128 (18.1)	< 0.001
Missing data	62 (4.4)	60 (7.8)	
Spleen involved			
Yes	362 (29.2)	208 (31.7)	0.29
Missing data	167 (11.9)	109 (14.2)	
Ki-67 > 30%			
Yes	596 (47.7)	610 (89.3)	< 0.001
Missing data	154 (10.9)	82 (10.7)	
HGB < 120 g/L			
Yes	341 (25.2)	170 (23.0)	0.27
Missing data	55 (3.9)	27 (3.5)	
LMR > 10			
Yes	193 (16.4)	67 (10.0)	0.0001
Missing data	96 (12.5)	10 (5.5)	
LDH > 245U/L			
Yes	292 (22.1)	243 (31.7)	
Missing data	81 (5.8)	40 (5.2)	< 0.0001
β2-MG ≥ 2.7 mg/L			
Yes	586 (44.6)	316 (43.1)	0.51
Missing data	92 (6.5)	32 (4.1)	
R-CHOP regimen			
Yes	862 (61.4)	543 (70.9)	< 0.0001
Missing data	164 (11.6)	77 (10.1)	
POD24			
Yes	287 (20.4)	151 (19.7)	0.70
Missing data	18 (1.2)	7 (0.9)	

Among the patients, 1403 (65%) and 765 (35%) were diagnosed as FL1-2 and FL3a, respectively. There were discrepancies in some baseline characteristics between these two subgroups. The percentage of elderly patients was higher in FL3a group, with 26% patients aged ≥ 60 years in the FL1-2 group (where the median age was 52 years) and 32% patients aged ≥ 60 years in FL3a (where the median age was 54 years, *P* = 0.003). Of note, compared to FL1-2 patients, high-grade FL3a patients presented more commonly with an elevated serum LDH level (32% versus 22%, *P* < 0.0001) and a higher Ki-67 index (> 30% in 89% cases versus 48% FL1-2 cases, *P* < 0.001). However, in FL3a, a lower proportion of patients presented with advanced stage (III/IV) diseases (71% cases versus 83% FL1-2 cases; *P* < 0.001), with a lower rate of BM infiltration (18% versus 34%; *P* < 0.001) and fewer patients having at least 4 involved LNs (59% versus 65%; *P* < 0.001). Other baseline clinical characteristics of FL3a patients in our cohort were comparable to FL1-2. These data suggested that FL3a patients presented distinctly different clinicopathological characteristics than low-grade FL1-2 patients.

### Treatment and responses

Eighty-nine percent of patients were treated with different frontline regimens with or without rituximab for varied cycles according to their clinical and disease features and in accordance with the Chinese Expert Consensus for FL. The treatment patterns and the responses are summarized in Supplementary Table 1. In brief, 688 FL3a patients and 1239 FL1-2 patients received the first-line treatment as follows: CHOP ± R regimens in 631 (92%) FL3a patients and 992 (80%) FL1-2 patients (*P* < 0.0001); lenalidomide + R (the R^2^ regimen) in 21 (3.1%) FL3a patients and 50 (4.0%) FL1-2 patients (*P* = 0.33); the BR regimen in 11 (1.6%) FL3a patients and 37 (2.9%) FL1-2 patients (*P* = 0.08); and the CVP ± R regimen in 8 (1.1%) FL3a patients and 24 (1.9%) FL1-2 patients (*P* = 0.27). Notably, 11% of FL1-2 patients (*n* = 136) were treated with the “watchful-waiting” approach; this proportion was markedly higher than the proportion treated with observation in the FL3a group of patients (2.4%, *P* < 0.0001).

**Fig. 1:**
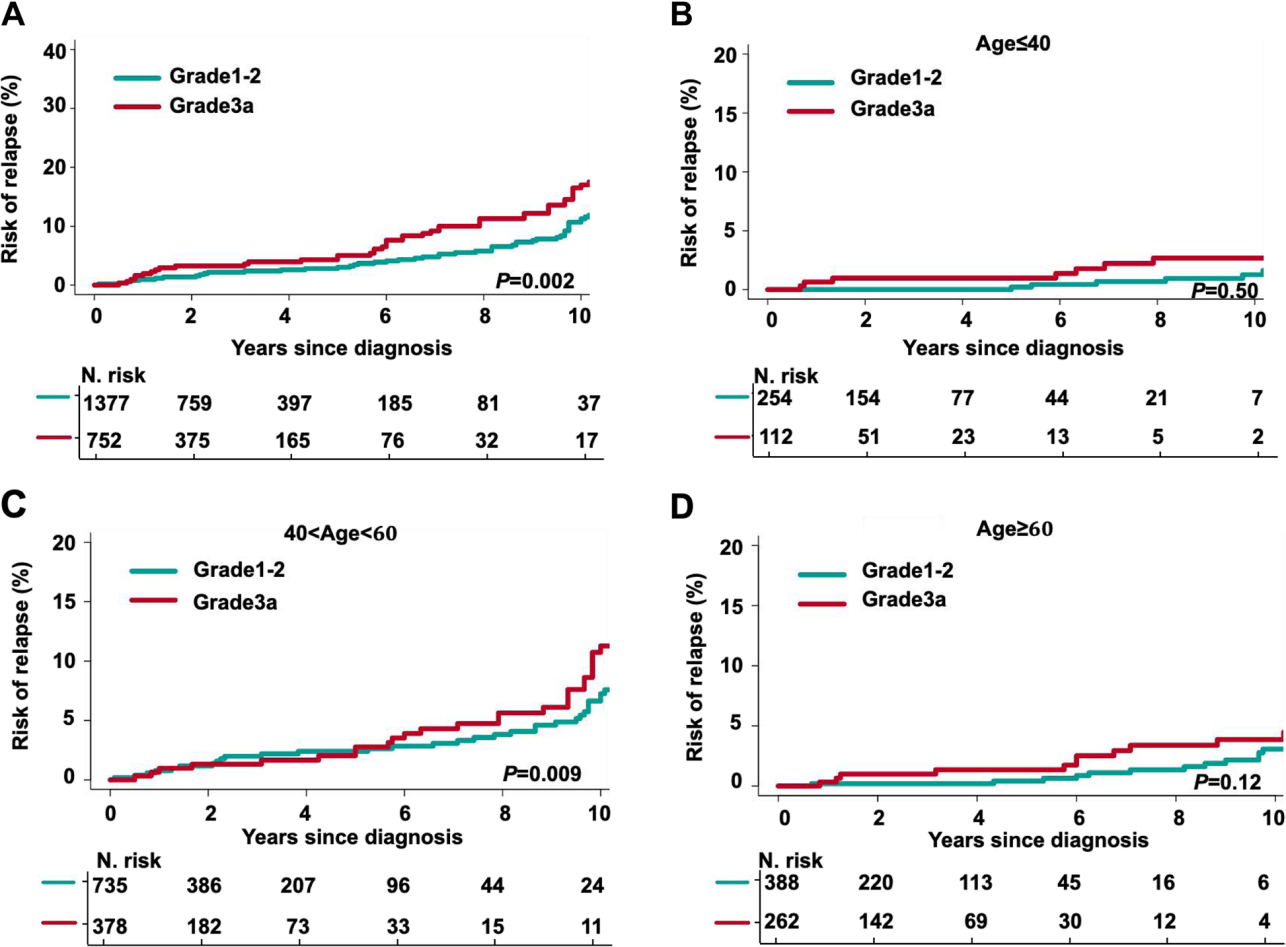
Comparison of the cumulative incidence of the competing risks of relapse between FL1-2 and FL3a. **A** Cumulative incidence of the competing risk for relapse in patients with FL grade 1–2 or 3a. **B**-**D** Cumulative incidence of the competing risk for relapse in patients with FL grade 1–2 or 3a aged ≤ 40 years **B**, between 40 and 60 years **C**, and aged > 60 years **D**

Among all treated patients, the CR rate to induction therapy was significantly higher in FL3a patients (57%) than in FL1-2 patients (48%, *P* < 0.001), while the overall response rate (ORR) was not significantly different between these two subgroups (79% versus 80%, *P* = 0.55). The higher CR rate in FL3a may have been because there were many more FL3a patients than FL1-2 patients who received CHOP ± R therapy. Although the CR rate to induction therapy was higher in FL3a, it was noted that FL3a patients had a higher risk of relapse (17.0% versus 11.2% at 10 years, *P* < 0.01, Fig. 1A), especially in patients aged between 40 and 60 years, with risk of relapse of 11.2% at 10 years (versus 7.2%, *P* < 0.01, Fig. 1C). No significant differences were found in younger or elderly patients (Fig. 1B and 1D). Among the patients with relapses, 13.7% (12/87) of FL3a patients showed histological transformation to aggressive lymphoma compared with 1.6% (2/106) of FL1-2 patients (*P* = 0.003), indicating that the transformation occurrence in FL patients, especially FL3a patients, is an important risk factor for relapse.

### Comparison of survival between FL1-2 and FL3a in the entire cohort

**Fig. 2:**
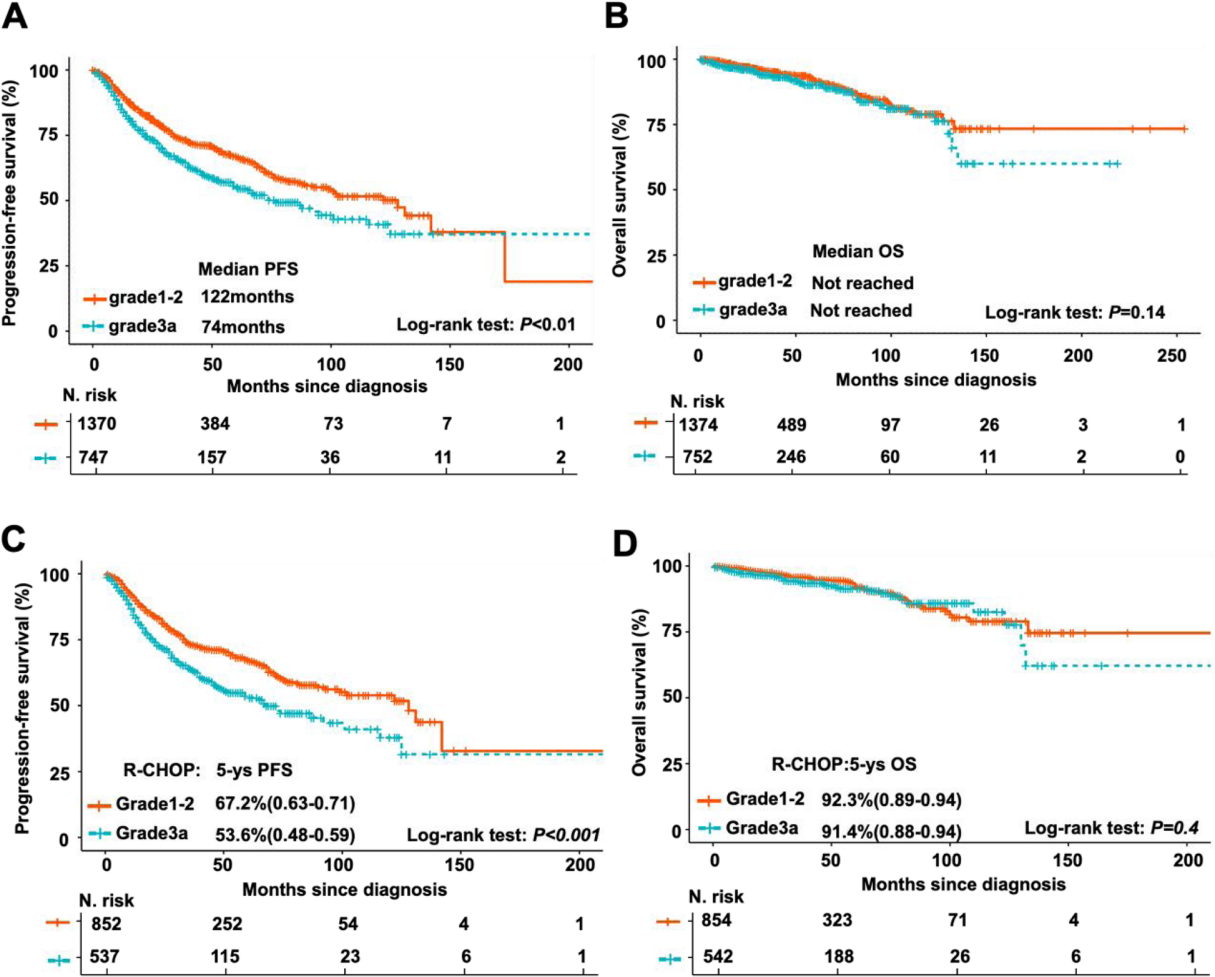
Comparison of clinical outcomes between patients with FL grade 1–2 and grade 3a. **A**-**B** Kaplan‒Meier curves of PFS (**A**) and OS (**B**) for FL1-2 and FL3a patients. The median PFS for FL1-2 patients (122 months, [95% CI: 44%-56%]), in comparison with FL3a patients (74 months; [95% CI: 45%-56%]); stratified P < 0.01. The median OS for FL1-2 and FL3a patients were both not reached. **C-D** Kaplan‒Meier curves of PFS (**C**) and OS (**D**) for FL1-2 and FL3a patients treated with R-CHOP regimen. 5-year PFS rate for FL1-2 patients who received R-CHOP therapy (67.2%, range 0.63–0.71), in comparison with FL3a patients who received R-CHOP therapy (53.6%, range 0.48–0.59); stratified *P* < 0.001. 5-year OS rate for FL1-2 patients who received R-CHOP therapy (92.3%, range 0.89–0.94), in comparison with FL3a patients who received R-CHOP therapy (91.4%, range 0.88–0.94); stratified *P* > 0.05

In all patients, the 5-year PFS and OS rates were 63% (95% CI 60.4–65.7; Supplementary Fig. 1A) and 90% (95% CI 89.2–92.6; Supplementary Fig. 1B), respectively. The differences regarding PFS, and OS between FL1-2 and FL3a patients were further evaluated. The median PFS for FL3a patients was 74 months (95% CI: 45%-56%), which was significantly lower than that for FL1-2 patients (122 months [95% CI: 44%-56%], *P* < 0.001, Fig. 2A), while the median values of OS for FL3a and FL1-2 patients were both not reached (Fig. 2B). These data indicated that FL3a patients had worse outcomes than FL1-2 patients. In this context, we further identified the prognostic factors involved in PFS and OS for FL3a patients, and a univariate Cox proportional hazard model was used to analyze the data. In the univariate analysis, advanced stage (III/IV) disease, involved LN regions > 4, bulky disease, HGB < 120 g/L and increased levels of LDH (> 1 × normal) were found to be significantly associated with inferior PFS. In addition, older age, B symptoms, and BM involvement contributed to unfavorable OS but not PFS (Table 2).

**Table 2:** Disease characteristic with univariate analysis in FL3a cohorts

C		PFS			OS	
	HR	95%CI	*P*	HR	95%CI	*P*
Age	1.005	0.99–1.01	0.27	1.03	1.01–1.05	0.001
Male	1.12	0.88–1.42	0.34	1.05	0.64–1.74	0.83
Rai stage III/IV	1.75	1.29–2.37	< 0.01	1.76	0.91–3.40	0.09
B symptom	1.20	0.84–1.70	0.31	2.06	1.10–3.87	0.02
β2-MG > 2.7 mg/L	1.11	0.87–1.42	0.39	2.66	1.56–4.55	< 0.001
Lymph nodes > 4	1.36	1.05–1.77	0.02	1.35	0.78–2.32	0.27
Extranodal disease	1.22	0.93–1.60	0.14	1.02	0.56–1.86	0.93
Bulky disease	1.39	1.04–1.86	0.02	1.26	0.70–2.26	0.42
Marrow involved	1.45	1.07–1.97	0.01	1.82	1.00–3.29	0.047
HGB < 120 g/L	1.47	1.11–1.93	< 0.01	1.53	1.12–1.64	< 0.01
Spleen involved	0.74	0.53–1.03	0.07	1.40	0.81–2.43	0.22
LDH > 245U/L	1.50	1.17–1.92	0.001	5.53	3.19–9.58	< 0.0001
LMR > 10	1.00	0.99–1.01	0.57	0.99	0.95–1.02	0.60
Ki-67 > 30%	0.82	0.56–1.21	0.33	0.79	0.33–1.87	0.60

### Outcomes of FL1-2 and FL3a patients receiving R-CHOP according to FLIPI and POD24

**Fig. 3:**
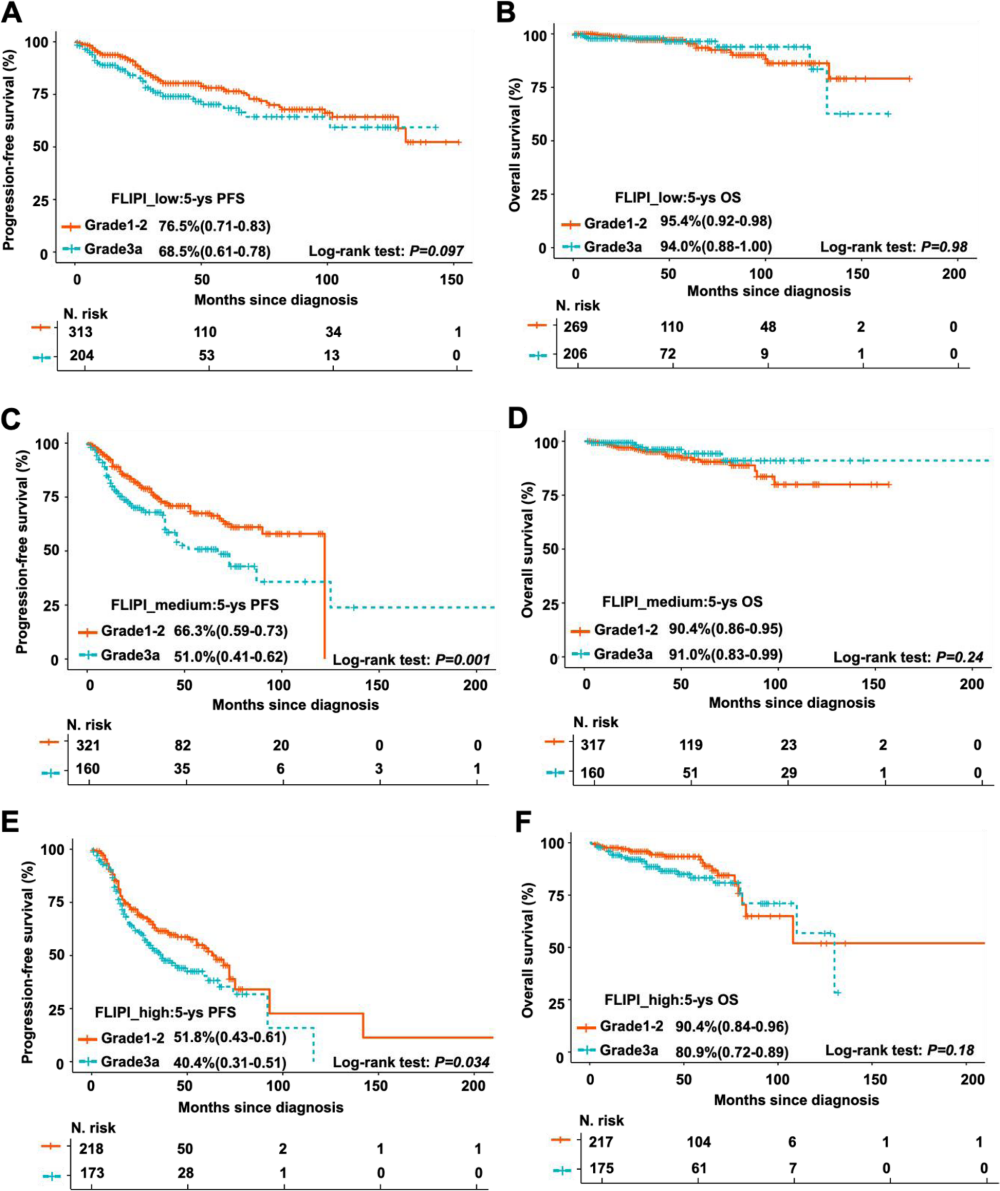
Comparison of clinical outcomes between patients with FL grade 1–2 and grade 3a stratified by FLIPI score. **A**-**B** Kaplan–Meier survival analysis of PFS **A** and OS **B** for FL1-2 and FL3a patients in FILPI low_risk group. 5-year PFS rate for FL1-2 patients with FLIPI 0–1(76.5%, range 0.71–0.83), in comparison with FL3a patients with FLIPI 0–1 (68.5%, range 0.61–0.78); stratified *P* > 0.05. 5-year OS rate for FL1-2 patients with FLIPI 0–1(95.4%, range 0.92–0.98), in comparison with FL3a patients with FLIPI 0–1 (94.0%, range 0.88–1.00); stratified *P* > 0.05. **C**-**D** Kaplan–Meier survival analysis of PFS **C** and OS **D** for FL1-2 and FL3a patients in FILPI intermediate_risk group. 5-year PFS rate for FL1-2 patients with FLIPI = 2 (66.3%, range 0.59–0.73), in comparison with FL3a patients with FLIPI = 2 (51.0%, range 0.41–0.62); stratified *P* = 0.001. 5-year OS rate for FL1-2 patients with FLIPI = 2 (90.4%, range 0.86–0.95), in comparison with FL3a patients with FLIPI = 2 (91.0%, range 0.83–0.99); stratified *P* > 0.05. **E–F** Kaplan–Meier survival analysis of PFS **E** and OS **F** for FL1-2 and FL3a patients in FILPI high_risk group. 5-year PFS rate for FL1-2 patients with FLIPI ≥ 3 (51.8%, range 0.43–0.61), in comparison with FL3a patients with FLIPI ≥ 3 (40.4%, range 0.31–0.51); stratified *P* < 0.05. 5-year OS rate for FL1-2 patients with FLIPI ≥ 3 (90.4%, range 0.84–0.96), in comparison with FL3a patients with FLIPI ≥ 3 (80.9%, range 0.72–0.89); stratified *P* > 0.05

**Fig. 4:**
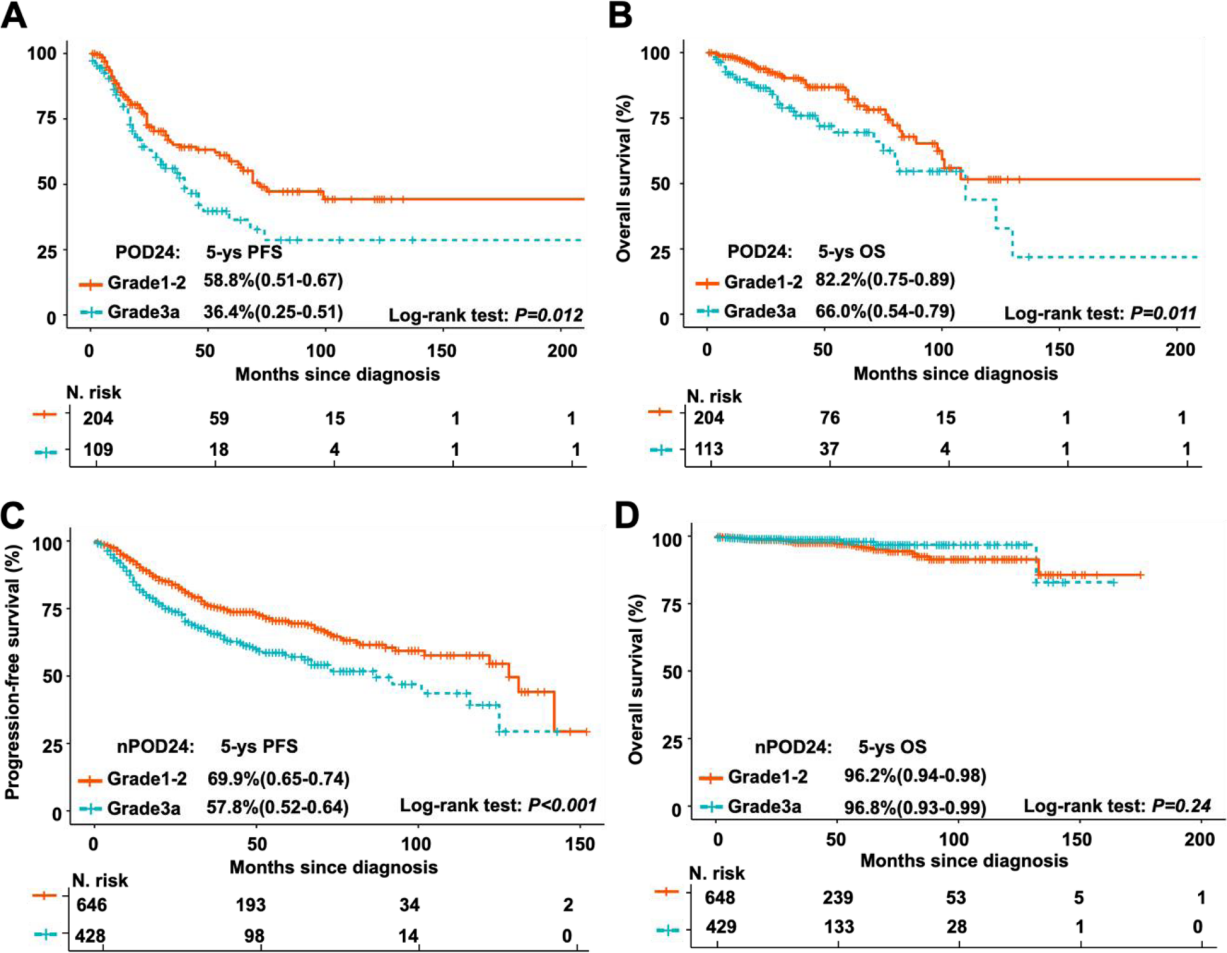
Comparison of clinical outcomes between patients with FL grade 1–2 and grade 3a in subsets with or without POD24. **A-B** Kaplan–Meier survival analysis of PFS **A** and OS **B** for FL1-2 and FL3a patients with POD24. 5-year PFS rate for FL1-2 patients with POD24 (58.8%, range 0.51–0.67), in comparison with FL3a patients with POD24 (36.4%, range 0.25–0.51); stratified *P* < 0.05. 5-year OS rate for FL1-2 patients with POD24 (82.2%, range 0.75–0.89), in comparison with FL3a patients with POD24 (66.0%, range 0.54–0.79); stratified *P* < 0.05. **C-D** Kaplan–Meier survival analysis of PFS **C** and OS **D** for FL1-2 and FL3a patients without POD24. 5-year PFS rate for FL1-2 patients without POD24 (69.9%, range 0.65–0.74), in comparison with FL3a patients without POD24 (57.8%, range 0.52–0.64); stratified *P* < 0.001. 5-year OS rate for FL1-2 patients without POD24 (96.2%, range 0.94–0.98), in comparison with FL3a patients without POD24 (96.8%, range 0.93–0.99); stratified *P* > 0.05

In our cohort, 862 (61.4%) of FL1-2 patients and 543 (70.9%) of FL3a patients received CHOP plus rituximab (R-CHOP), which was the most used regimen as front-line therapy (Table 1). In this subgroup analysis, FL3a patients also exhibited shroter PFS (Fig. 2C), but not OS (Fig. 2D), than FL1-2 patients. Moreover, differences in patient outcomes were evaluated according to FLIPI risk stratification as well as in patients with or without progression within 24 months (POD24). In the low-risk patients defined by FLIPI, the survival curves of FL1-2 and FL3a patients were overlapped without significant differences (Fig. 3A, 5-year PFS of 76.5% versus 68.5%,* P* = 0.097; Fig. 3B, 5-year OS of 95.4% versus 94%,* P* = 0.98). In the FLIPI intermediate- or high-risk groups, FL3a patients had shorter PFS than FL1-2 patients (Fig. 3C, 5-year PFS of 66.3% versus 51.0%,* P* = 0.001; Fig. 3E, 5-year PFS of 51.8% versus 40.4%,* P* = 0.034), but similar OS between these two groups (Fig. 3D, 5-year OS of 90.4% versus 91.0%,* P* = 0.24; Fig. 3F, 5-year OS of 90.4% versus 80.9%,* P* = 0.18). POD24, a well-established dismal prognostic predictor of FL, was observed in 287 (20.4%) FL1-2 cases and 151 (19.7%) FL3a cases (Table 1). FL3a patients with POD24 had shorter PFS (Fig. 4A, 5-year PFS of 58.8% versus 36.4%,* P* = 0.012) and OS (Fig. 4B, with 5-year OS of 82.2% versus 66.0%,* P* = 0.011) than FL1-2 patients. However, although FL3a patients also had shorter PFS than FL1-2 patients in patients without POD24 (Fig. 4C, 5-year PFS of 69.9% versus 57.8%,* P* < 0.001), there were no significant differences in OS between these two groups (Fig. 4D, 5-year OS of 96.2% versus 96.8%,* P* = 0.24).

**Fig. 5:**
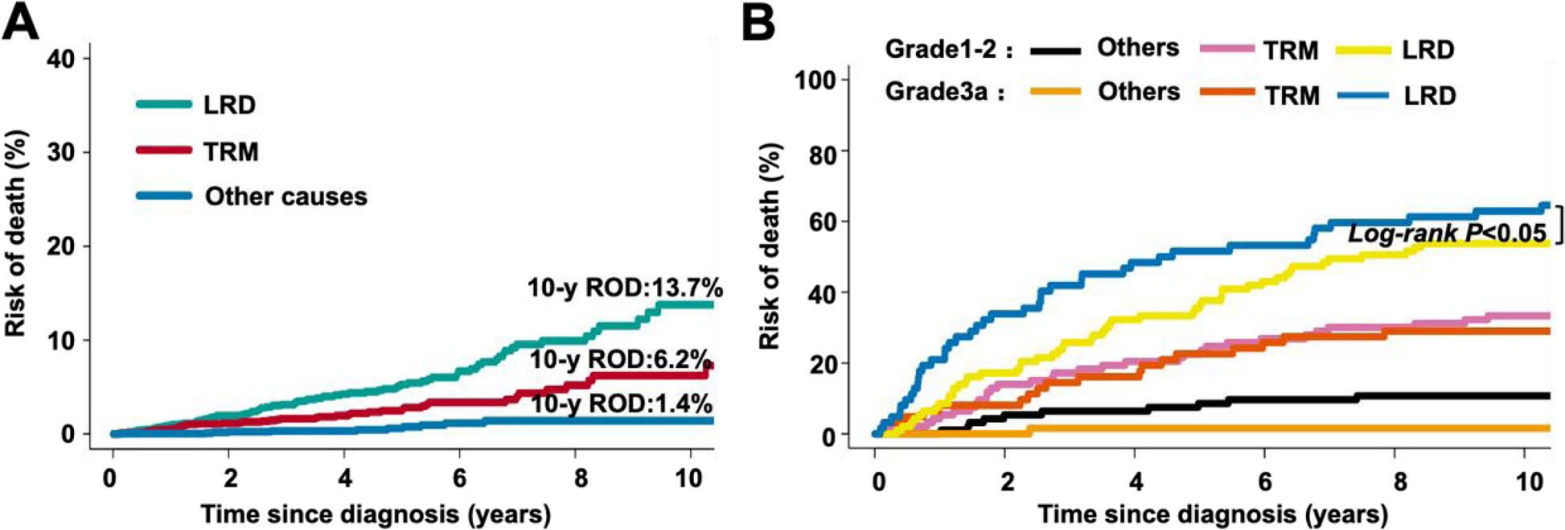
Cumulative incidence of the competing risks of COD. **A** Cumulative incidence of each COD. **B-D** Cumulative incidence of each COD for FL1-2 and FL3a patients

### Comparison of causes of death between FL1-2 and FL3a patients

With a median follow-up of 38 months (range, 1–246 months), by the end of follow-up, 7% of 2168 FL (FL1-2 and FL3a) patients had died from all causes. Among all patients, lymphoma-related deaths (LRDs) were the most common cause of death with 5-year and 10-year cumulative incidences of 5.2% and 13.7%, respectively, followed by treatment-related mortality (2.4% and 6.2%) and other causes (0.6% and 1.4%; Fig. 5A), respectively. In the subgroup analysis, LRD and TRM also represented two predominant causes of death in FL1-2 and FL3a patients. However, the 4-, 6- and 10-year cumulative incidence rates of LRDs for FL3a patients were 48%, 53%, and 62%, while the 4-, 6- and 10-year cumulative incidence rates of LRDs for FL1-2 patients were 32%, 43% and 53%, respectively (*P* < 0.05). In addition, there was no difference in the cumulative incidence rates of TRM between FL1-2 and FL3a (Fig. 5B).

### Histological transformation between FL1-2 and FL3a

**Fig. 6:**
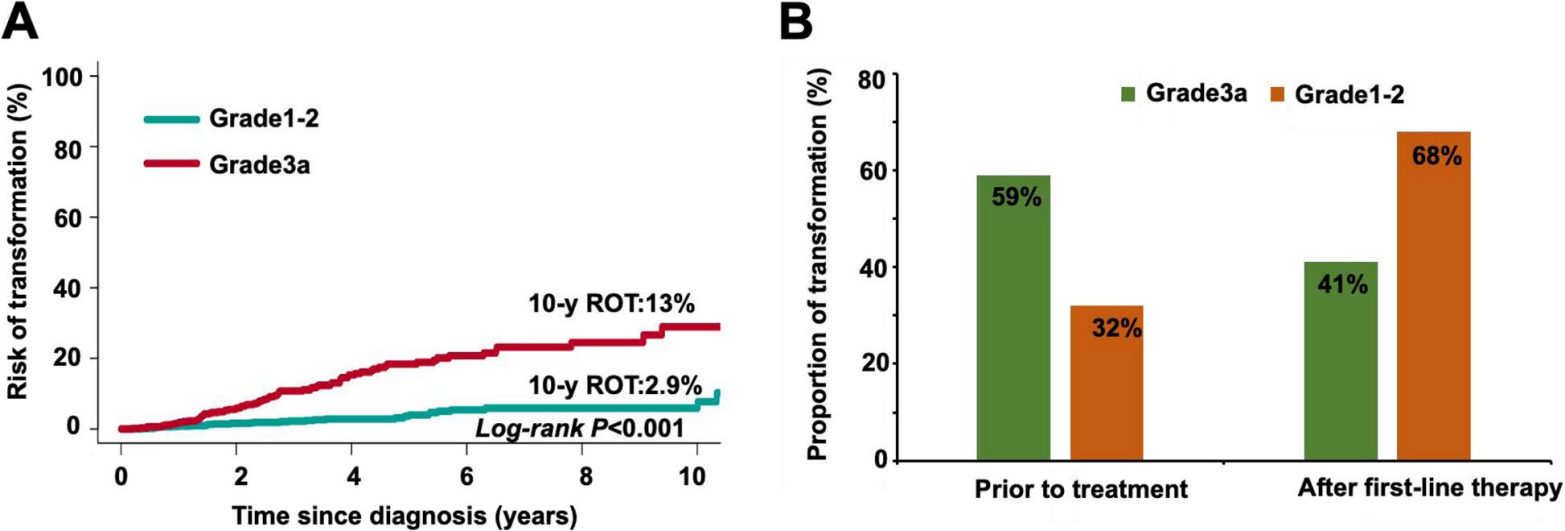
Comparison of the cumulative incidence of the competing risks of transformation between FL1-2 and FL3a. **A** Cumulative incidence of the competing risk for transformation in patients with FL grade 1–2 or 3a. **B** The proportion of patients with transformation prior to therapy and after therapy in FL1-2 and FL3a

**Fig. 7:**
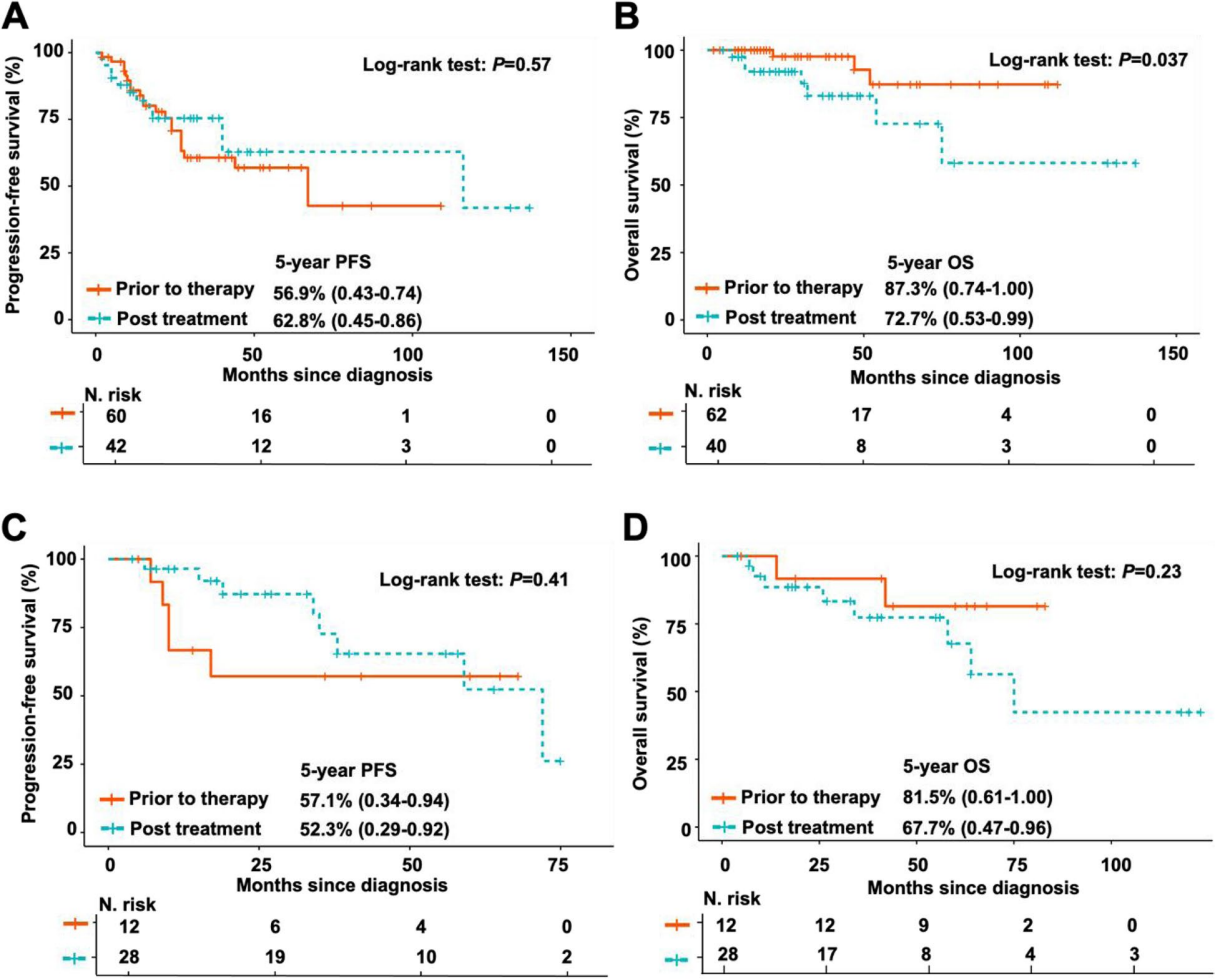
The prognostic influence of histological transformation prior to and after therapy in FL1-2 and FL3a patients. **A**-**B** Kaplan‒Meier curves of PFS **A** and OS **B** for FL3a patients with HT prior to therapy and after therapy. 5-year PFS rate for FL3a patients with HT prior to therapy (56.9%, range 0.43–0.74), in comparison with FL3a patients with HT after therapy (62.8%, range 0.45–0.86); stratified *P* > 0.05. 5-year OS rate for FL3a patients with HT prior to therapy (87.3%, range 0.74–1.00), in comparison with FL3a patients with HT after therapy (72.7%, range 0.53–0.99); stratified *P* < 0.05. **C**-**D** Kaplan‒Meier curves of PFS **C** and OS **D** for FL1-2 patients with HT prior to therapy and after therapy. 5-year PFS rate for FL1-2 patients with HT prior to therapy (57.1%, range 0.34–0.94), in comparison with FL1-2 patients with HT after therapy (52.3%, range 0.29–0.92); stratified *P* > 0.05. 5-year OS rate for FL1-2 patients with HT prior to therapy (81.5%, range 0.61–1.00), in comparison with FL1-2 patients with HT after therapy (67.7%, range 0.47–0.96); stratified *P* > 0.05

Histologic transformation (HT) was observed in 6.7% (43 FL1-2 and 103 FL3a) of 2168 patients. Of the transformed FL (t-FL) patients, 99% of cases transformed to DLBCL except for one patient whose disease transformed to lymphoblastic lymphoma. Competing risk analysis revealed that the cumulative rates of HT at 5 and 10 years were 10% and 13% for FL3a patients, respectively; these rates were markedly higher than those for FL1-2 patients (2.1% and 2.9%, *P* < 0.001, Fig. 6A). Furthermore, transformation occurred prior to treatment in 14 (32%) FL1-2 cases or after first-line therapy in 29 (68%) FL1-2 cases, while the proportion of patients with HT prior to treatment was higher (59%) and the proportion of patients with HT after therapy was lower (41%) in FL3a cases (*P* = 0.005, Fig. 6B). In the FL3a cohort, the 5-year OS rate of patients with t-FL who transformed prior to treatment was 87%, which was higher than that for those who transformed after treatment (72%, *P* = 0.037; Fig. 7B). However, there was no difference in the 5-year PFS rate between these two groups (*P* = 0.79, Fig. 7A). In contrast, there was no difference in the 5-year PFS and OS rates between patients who transformed prior to treatment and those who transformed after treatment in patients with FL1-2 (*P* = 0.41 and *P* = 0.23, Fig. 7C and 7D).

## Discussion

There is no certainty regarding whether FL3a should be distinguished from FL1-2 before therapy is initiated [21]. To the best of our knowledge, this study is the largest to compare the demographic and disease characteristics, treatment patterns, clinical outcomes, and mortality related to FL1-2 and FL3a in a real-world population-based study in China. In our cohort, FL3a accounted for 30% of all FL patients, which is obviously lower than the proportion attributable to FL1-2. This observation is consistent with other published studies [6, 8, 17]. Although a previous study showed that FL3a shares similarities with FL1-2 in immunohistochemical characteristics [22], survival differences actually exist in relationship to histological grading, and no concordant agreements have been reached regarding relapse and prognosis in FL3a and FL1-2 patients [23–25]. In a recent study based on surveillance, epidemiology and end results (SEER) data performed by Naik et al., which included 39,925 FL patients with nodal FL, the effect of histological grading on treatment outcomes and prognosis was compared [26]. Even though the study determined that FL3 had a more aggressive behavior and worse prognosis than low-grade FL1-2, FL3b cases were not removed in that study since it was not only for FL3a. Additionally, but there was also no discussion of the differences in clinical features at diagnosis on account of histological grading. Unlike the difference in OS between FL3 and FL1-2 in that study, patients with FL3a in our cohort had shorter PFS than FL1-2 patients but not shorter OS.

For clinicians, several questions remain unanswered: Are there any differences in clinical characteristics and behaviors in FL3a patients compared with FL1-2 patients? Should different therapeutic strategies be applied for the two categories [9, 27]? For the first question, the 2168 follicular lymphoma cases of different histologic grades were analyzed in this study. In comparison with FL1-2 patients, FL3a patients displayed several different demographic characteristics, including the following: a) although the median age of FL3a patients at diagnosis was 54 years and comparable with that of FL1-2, elderly patients (> 60 years) represented 32% of FL3a cases, a higher than that observed for the FL1-2 group; b) FL3a patients had elevated baseline serum LDH activity, which was associated with an inferior PFS in univariate analysis, similar to that reported in previous studies; c) FL3a had a higher cell proliferation rate by Ki-67 staining (Ki-67 > 30% in 89% FL3a patients vs. 48% FL1-2 patients). Thus, we speculate that the survival difference between FL3a patients and FL1-2 patients may be attributed to different biological behaviors in the two lymphoma categories.

This study covering the range of years from 2000 to 2020 revealed the evolution of FL treatment in China, particularly providing comprehensive evidence supporting the groundbreaking effects of rituximab on outcomes for Chinese FL patients. The bulk of evidence supports the idea that the addition of rituximab to conventional chemotherapies represents a critical independent factor for favorable outcomes in FL patients [28, 29]. In this large cohort of Chinese FL patients, most of the patients received CHOP without rituximab as the induction regimen until 2010, while the use of rituximab sharply increased afterward, likely due to confirmation of the superb effect of the R-CHOP regimen on the survival of FL patients by numerous clinical trials in China, which also led to updating the Chinese guidelines for clinical practice [30]. Various international guidelines for clinical practice do not differentiate the use of bendamustine + rituximab (BR) and CHOP + rituximab (R-CHOP) as frontline therapy in the treatment of FL. However, low-grade FL patients are often treated with the BR regimen by virtue of its lower toxicity [18, 31]. It remains controversial which treatment would bring long-term benefits (including PFS and OS) to FL3a patients. In our cohort, CHOP with or without rituximab was the most common regimen, with only 1.6% of FL3a patients receiving the BR regimen, likely due to the unavailability of bendamustine in China until 2019. Thus, caution needs to be taken when comparing the effects of BR with other regimens on the outcome of FL3a patients.

In this cohort of Chinese FL patients, the 5-year PFS and OS rates were 63% and 90%, respectively, similar to those reported in Western countries [32]. Lymphoma progression (LRD) is the leading cause of death in the first decade after diagnosis as reported in recent cohort studies in Western countries [33]. This was particularly true for patients who did not achieve event-free survival within 24 months (EFS24), as well as for those with high FLIPI scores or transformed FL (t-FL). In our cohort, LRD represented the most common cause of death (60%), consistent with the results obtained from a pooled analysis of two independent studies involving FL1-3a patients [33]. In a recent population-based study enrolling 1928 patients, higher mortality was reported in elderly FL patients (> 65 years) [34]. In this context, we observed that the LRD and TRM were the primary causes of death in FL3a patients. Moreover, the LRD rate was also higher in FL3a patients than in FL1-2 patients. Therefore, both disease progression (LRD) and treatment (TRM) contribute to high mortality in FL3a patients; these causes of death, therefore, remain a major challenge in the management of FL3a patients.

Despite the remarkable improvement of OS in FL patients in the era of rituximab, the outcome for FL patients with HT remains dismal with high risk of death [35, 36]. HT to aggressive lymphoma (t-FL), which occurs in approximately 3% of FL patients per year with no plateau [37], has a well-established correlation with worse outcomes than nontransformed FL (nt-FL). In our research reported previously [30], the cumulative incidence of mortality for patients with t-FL was greater than that for those with nt-FL. Further analysis of patients with t-FL suggested that the cumulative incidence of LRD was significantly higher than that of death unrelated to lymphoma, consistent with the results reported by population-based studies in the United States and France. Comparable results were obtained in patients with nt-FL. These observations further confirmed HT as a major cause of LRD in FL patients, likely across different populations. Of note, the cumulative incidence of transformation in FL3a was much higher than that in FL1-2. In addition, there was a marked difference in OS between FL3a patients with HT prior to therapy and after therapy. Mechanistically, clonal evolution stemming from the disease itself or chemotherapy may contribute to the adverse effects of HT after therapy on the outcomes in FL3a patients.

## Conclusions

In summary, FL3a patients are characterized by more elderly patients, elevated LDH, and a higher Ki-67 index and have worse prognosis than FL1-2 patients in the Chinese population. FL3a patients were also found to have a higher LRD, which may be associated with the aggressive behavior of their disease. In addition, patients with posttreatment HT had a higher risk of mortality than those with HT prior to therapy, emphasizing the adverse role of HT (particularly that occurring after treatment) in the outcome of FL3a patients. Although this work provided strong evidence that FL3a should be distinguished from low-grade FL in terms of clinical characteristics and outcomes, we contend that additional prospective studies are needed to further characterize these outcomes and guide optimal treatment decisions for FL3a patients.

## Supplementary Information

**Additional file 1:** **Supplementary Figure S1.** Clinical outcomes in Chinese FL patients. (A-B) Kaplan‒Meier curves of PFS (A) and OS (B) for all FL patients. 5-year PFS rate and OS rate for Chinese FL patients were 63% (range: 0.60-0.65) and 90% (range: 0.89-0.92), respectively.

**Additional file 2:** **Supplementary Table 1.** Treatment regimens and effect evaluation in FL1-2 and FL3a patients.

## Data Availability

All relevant data and materials have been involved in the article. Further inquiries can be directed to the corresponding authors.
