# Supplementary figures and images for: Differences in clinical characteristics and outcomes between patients with grade 3a and grades 1–2 follicular lymphoma: a real-world multicenter study
Source: Biomark Res. 2023 Feb 6;11:16. doi: 10.1186/s40364-023-00462-z (PMC9901161; doi:10.1186/s40364-023-00462-z)

# Supplementary Figure 1

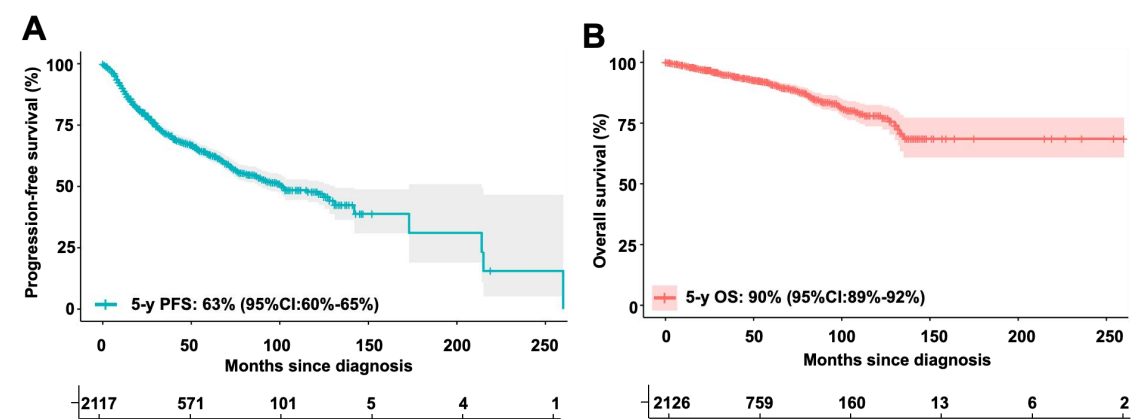

Supplement: Supplementary file 1 — Additional file 1: Supplementary Figure S1. Clinical outcomes in Chinese FL patients. (A-B) Kaplan‒Meier curves of PFS (A) and OS (B) for all FL patients. 5-year PFS rate and OS rate for Chinese FL patients were 63% (range: 0.60-0.65) and 90% (range: 0.89-0.92), respectively. [file 40364_2023_462_MOESM1_ESM.pdf]
