# Supplementary material for: Differences in clinical characteristics and outcomes between patients with grade 3a and grades 1–2 follicular lymphoma: a real-world multicenter study
Source: Biomark Res. 2023 Feb 6;11:16. doi: 10.1186/s40364-023-00462-z (PMC9901161; doi:10.1186/s40364-023-00462-z)
Supplement: Supplementary file 2 — Additional file 2: Supplementary Table 1. Treatment regimens and effect evaluation in FL1-2 and FL3a patients. [file 40364_2023_462_MOESM2_ESM.docx]

**Supplementary table 1. Treatment regimens and effect evaluation in FL1-2 and FL3a patients**

|  | **FL1-2** | **FL3A** | ***P* value** |
| --- | --- | --- | --- |
| **Treatment (n, %)** | **N=1239** | **N=688** |  |
| CHOP±R | 992 (80) | 631(92) | <0.0001 |
| CVP±R | 24 (1.9) | 8 (1.1) | 0.27 |
| R^2^ | 50 (4.0) | 21 (3.0) | 0.33 |
| BR | 37 (3.0) | 11 (1.6) | 0.08 |
| Watch and wait | 136 (11) | 17 (2.4) | <0.0001 |
| **Response (n, %)** |  |  |  |
| CR | 585(47.2) | 396 (57.5) | <0.001 |
| PR | 411 (33.1) | 148 (21.5) | <0.0001 |
| relapse | 106 (8.5) | 87 (12.6) | 0.005 |
